# End-of-Year Reflections and Looking to the Future After 20 Years of Scholarly Publishing in Public Health Research, Evaluation, and Practice

**DOI:** 10.5888/pcd21.240503

**Published:** 2024-12-12

**Authors:** Leonard Jack

**Affiliations:** 1 Preventing Chronic Disease, Office of the Director, National Center for Chronic Disease Prevention and Health Promotion, Centers for Disease Control and Prevention, Atlanta, Georgia

**Figure:**
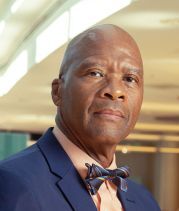
Leonard Jack, Jr, PhD, MSc

The past 20 years have been an extraordinary journey for *Preventing Chronic Disease*, marked by increases in its impact factor resulting from the dissemination of thousands of high-quality peer-reviewed articles from around the world. As public health practice expands to address emerging health issues, the journal will continue to serve as a valuable and respected resource to public health experts working in state and local health departments, academic institutions, health systems, community settings, and beyond.

Since its inception in 2004, PCD has expanded its topics of interest and the size of its volunteer groups — the editorial board, associate editors, Statistics Review Committee — and more recently, its Student Scientific Writing and Review Training Committee. We recognize that our path forward will require building on the contributions of the public health experts who have volunteered hundreds of hours of time to bring expertise in the use of various research methodologies, evaluation approaches, evidence-based approaches to improve population health, statistical analyses, and strategies to maximize community and partnership engagement.

In 2024, the journal published 7 collections that address a range of important public health topics:

- **Mapping Chronic Disease in the United States.** Seven peer-reviewed articles use geospatial maps to depict complex information in an easy-to-understand format on patterns, relationships, and levels of disease or behavior in geographic areas across the US (1).
- **From Data to Action: National, State & Local Efforts to End Menthol and Other Flavored Commercial Tobacco Product Use.** The 10 peer-reviewed articles in this collection enhance understanding of public health’s role in reducing tobacco-related diseases and deaths, highlight surveillance data on menthol and other flavored tobacco, and provide examples of state and local activities implemented in this area (2).
- **Policy, Systems, and Environmental Approaches in Chronic Disease Research and Practice.** Seven peer-reviewed articles explore how research, surveillance, and evaluation can be used to advance the use and application of policy, systems, and environmental approaches in public health (3).
- **Public Health Actions to Reduce the Burden of Asthma.** This collection consists of 10 peer-reviewed articles that highlight the history of asthma control programs funded by the Centers for Disease Control and Prevention in the US, describe the burden of asthma among those most affected, and use examples to illustrate the development, implementation, and evaluation of asthma-related interventions to improve asthma control (4).
- ***Preventing Chronic Disease* 20th Anniversary Collection.** This collection features 32 seminal articles published in the journal over the past 20 years. Although it is impossible to feature the many articles that fall into this category, those included in this collection showcase the journal’s progress over the years and include articles that received a high number of citations, were most talked about in the news media or on social media, or described PCD’s accomplishments and future directions (5).
- **Advancing Chronic Disease Data Modernization Enhancements to Meet Current and Future Public Health Challenges.** Nine peer-reviewed articles in this collection highlight advancements in chronic disease surveillance. They emphasize the importance of collaboration and coordination among partners, describe solutions that predominantly use data from electronic health record systems, underscore the challenges of chronic disease surveillance, and discuss future considerations (6).
- **2024 Student Paper Contest.** For this collection of 9 peer-reviewed articles, students at the high school, undergraduate, graduate, and other levels served as first authors. Articles address topics such as screening for colorectal cancer, mental health services among children with mental health disorders, lifestyle intervention programs for uninsured Spanish-speaking immigrants, racial and ethnic disparities in food access and dietary intake, and hospital readmissions for heart failure, COPD, myocardial infarction, and stroke (7).

## Emerging Topics in 2025

We look forward in 2025 to an exciting year of disseminating rich content that will position the journal to reach a larger audience, continue to improve its domestic and global impact, build the future workforce by enhancing skills and abilities among students to conduct and publish quality research, and secure the participation of a range of expertise among the journal’s volunteers. With the journal’s two-decade commitment to excellence, we anticipate releasing the following collections in 2025:

- **Emerging Issues in Public Health Research and Program Strategies for Diabetes Prevention and Management: A Blueprint for Action.** This collection will highlight recent efforts by the National Diabetes Prevention Program to prevent or delay the onset of type 2 diabetes among those identified at higher risk, the development of effective diabetes self-management education and support services to reduce the risk of complications among people with diabetes, and recognition of the critical role that social determinants of health drivers play in disparities in the risk of type 2 diabetes and its complications.
- **Community Engagement and Population Health: From Practice to Evaluation.** Articles in this collection will reflect on how and where community engagement has successfully occurred, including information on populations engaged, their geographical locations, types of partners involved, community engagement strategies used, and advances in measuring community engagement.
- **Comorbidity Causes, Health Implications, and Multisystem Approaches to Treatment and Care.** Articles in this collection may cover such topics as the prevalence of multimorbidities among racial and ethnic groups, the effect of lack of access to health care on the treatment and care of comorbidities across the lifespan, and clinical or community-delivered interventions that address health behaviors that increase the risk of developing coexisting conditions.
- **American Indian and Alaska Native COVID-19 and Other Leading Causes of Mortality in 2020.** These articles will describe methods for linking data from the National Death Index with the Indian Health Service patient registration database for the correction of racial misclassification of vital statistics data among American Indian and Alaska Native populations. They will also provide data on COVID-19–related deaths and leading causes of deaths among this population in 2020.
- **Rural Health Disparities: Contemporary Solutions for Persistent Rural Public Health Challenges.** Public health challenges have been documented in rural areas and remain persistent (8). These challenges include limited access to health care, excessive tobacco use in lower-income counties, limited physical activity, behavioral and mental health conditions, and major chronic diseases. The goal of this collection is to offer concrete solutions to these challenges.
- **Social Determinants of Health**. These original research articles will use data from the Social Determinants of Health module of the 2022 Behavioral Risk Factor Surveillance System to highlight the intersectional relationships among social determinants of health, health-related social needs, and chronic diseases among adults, thus furthering the mission of the National Center for Chronic Disease Prevention and Health Promotion’s mission to prevent chronic diseases and promote the health and well-being of communities.
- **Community Health Workers for COVID Response and Resilient Communities.** The Centers for Disease Control and Prevention’s National Center for Chronic Disease Prevention and Health Promotion launched the Community Health Workers for COVID Response and Resilient Communities in 2021 (9). Peer-reviewed articles in this collection will describe how this initiative provided technical assistance to 67 organizations across the US to support the training and deployment of community health workers and resulted in strengthening community capacity and resilience to respond during the COVID-19 crisis.
- **2025 Student Paper Collection.** This collection will feature student research related to the prevention, screening, surveillance, and population-based intervention of chronic diseases, including arthritis, asthma, cancer, depression, diabetes, obesity, and cardiovascular disease, as well as COVID-19 (10). In addition, the collection will feature student essays discussing how to approach persistent or emerging public health challenges with new solutions (11).

## Conclusion

The well-established foundation in scholarly publishing that the past 20 years has provided us will enable the journal to continue innovating its day-to-day operations to move into the next decade of advances in its global standing and ranking. Our editorial board members will continue to provide impeccable guidance and direction. Our associate editors and peer reviewers, through their provision of hundreds of hours of technical expertise to authors and the journal, will assist us in selecting articles of even higher quality. Members of the journal’s Statistics Review Committee will continue adding to PCD’s future successes by providing specialized areas of expertise and by critiquing statistical methods reported in manuscripts submitted. As editor in chief, I believe it is important to conclude this year’s final Editor-in-Chief’s column by acknowledging PCD’s hard-working staff. These staff members are at the core of the journal’s past and future success. Most of our staff members have been with the journal for its entire 20-year existence. In fact, PCD’s staff has a combined 225 years of experience working at the journal.

PCD has an exciting future. We have repeatedly stated over the years that the journal’s success is due to the unwavering support from so many groups and individuals. Thank you for helping the journal to make an indelible mark on public health research, evaluation, and practice.
